# Beyond the scope and the glue: update on evaluation and management of gastric varices

**DOI:** 10.1186/s12876-020-01513-7

**Published:** 2020-10-30

**Authors:** Cyriac Abby Philips, Rizwan Ahamed, Sasidharan Rajesh, Tom George, Meera Mohanan, Philip Augustine

**Affiliations:** 1 The Liver Unit and Monarch Liver Laboratory, Cochin Gastroenterology Group, Ernakulam Medical Center, Kochi, Kerala 682028 India; 2 Gastroenterology and Advanced G.I Endoscopy, Cochin Gastroenterology Group, Ernakulam Medical Center, Kochi, Kerala 682028 India; 3 Division of Hepatobiliary Interventional Radiology, Cochin Gastroenterology Group, Ernakulam Medical Center, Kochi, Kerala 682028 India; 4 Anaesthesia and Critical Care, Cochin Gastroenterology Group, Ernakulam Medical Center, Kochi, Kerala 682028 India

**Keywords:** Endoscopy, EUS, Bleeding, Cirrhosis, Portal hypertension, Portal vein, TIPS, BRTO, CARTO, PARTO

## Abstract

Gastric varices are encountered less frequently than esophageal varices. Nonetheless, gastric variceal bleeding is more severe and associated with worse outcomes. Conventionally, gastric varices have been described based on the location and extent and endoscopic treatments offered based on these descriptions. With improved understanding of portal hypertension and the dynamic physiology of collateral circulation, gastric variceal classification has been refined to include inflow and outflow based hemodynamic pathways. These have led to an improvement in the management of gastric variceal disease through newer modalities of treatment such as endoscopic ultrasound-guided glue-coiling combination therapy and the emergence of highly effective endovascular treatments such as shunt and variceal complex embolization with or without transjugular intrahepatic portosystemic shunt (TIPS) placement in patients who are deemed ‘difficult’ to manage the traditional way. Furthermore, the decisions regarding TIPS and additional endovascular procedures in patients with gastric variceal bleeding have changed after the emergence of ‘portal hypertension theories’ of proximity, throughput, and recruitment. The hemodynamic classification, grounded on novel theories and its cognizance, can help in identifying patients at baseline, in whom conventional treatment could fail. In this exhaustive review, we discuss the conventional and hemodynamic diagnosis of gastric varices concerning new classifications; explore and illustrate new ‘portal hypertension theories’ of gastric variceal disease and corresponding management and shed light on current evidence-based treatments through a ‘new’ algorithmic approach, established on hemodynamic physiology of gastric varices.

## Disease definition and epidemiology

Portal hypertension (PH) is a syndrome characterized by the formation of portosystemic collaterals in the presence or absence of cirrhosis. The cardinal feature of PH is the formation of varices, which are dilated pre-existing or newly formed portosystemic venous channels, commonly found in esophageal and gastric regions that at risk for gastrointestinal bleeding. The prevalence of varices in cirrhosis can range from 40% in patients with Child–Pugh class A to approximately 85% in those in Child–Pugh class C [1, 2]. Gastric varices (GV; commonly classified using the Sarin system) are less common than esophageal varices with an incidence between 2 and 20%. As a general rule, GV is noted in one out of every five patients with cirrhosis [3]. The cumulative risk of bleeding from GVs 16%, 36%, and 44% at one, three, and five years follow up respectively, in patients without bleeding at diagnosis. Another large study showed that the cumulative bleeding was 4.8%, 19.9%, and 23.2%, respectively [3, 4]. Among GV, most common is Type 1 gastroesophageal varices (GOV), representing 70% of all GV, followed by Type 2 GOV in 21%.The highest risk of bleeding is associated with Type 1 IGV followed by Type 2 GOV. Acute variceal bleeding is a severe complication of cirrhosis that can lead to death in one-third of affected patients at 6 weeks. Even though only 10–30% of variceal bleeds are related to GV, it is associated with higher transfusion requirements, uncontrolled bleeding, rebleeding, and death. GVs bleed less frequently than esophageal varices but tend to bleed more severely. The severity of PH, as measured by the hepatic venous pressure gradient (HVPG), is beneficial in determining the risk of bleeding, rebleeding, and uncontrolled bleeding from esophageal varices. However, in GVs, the risk of bleeding is not entirely dependent on the degree of PH, but more related to the size of varices, the wall tension, and presence of red color signs over varix [5, 6]. A thorough understanding of the anatomy and pathophysiology of pertinent collateral pathways is required to decide on the best possible treatment option(s) for bleeding from GVs, beyond current recommendations.

## Diagnosis

### Endoscopic evaluation-based diagnosis and classification

Stadelmann, in 1913 described the formation of GVs in association with PH. Esophago-gastro-duodenoscopy, the gold standard test for diagnosing gastroesophageal varices classifies them according to size as small (< 5 mm) or large (> 5 mm) [7]. Endoscopic ultrasonography (EUS) can additionally assess collateral pathway anatomy and identify of perforating veins which improves treatment response monitoring in real-time [5, 7, 8]. Nonetheless, EUS is not recommended as the primary tool for assessment due to limited availability and need for expertise. Capsule endoscopy in grading and diagnosis of esophageal and GV has an accuracy of 90% with pooled sensitivity and specificity of 83% and 85%, respectively [9, 10]. However, it is limited to patients unwilling for conventional invasive procedures. Liver stiffness < 20 kPa along with platelet count of > 150,000 per microliter is associated with < 5% chance of having high-risk varices. However, this non-invasive measurement is not validated in GVs [11]. Initially, GV was classified into F1—mild, F2—moderate and F3—severe, ‘forms’ (Choi classification) and after that into those associated with splenic vein thrombosis and those associated with cirrhosis or its absence thereof. Hoskins and Johnson in 1988 provided the first full descriptive classification of GV, into three types, based on the relation and extension of GV with the esophagus. Hashizume based variceal descriptions on the underlying vascular anatomy and presence of red color signs. Sarin classification was based on the location that aid in the choice of therapy. The Sarin GV classification is the most commonly followed, which is also endorsed by the Baveno [3, 6, 7]. Multiple other systems for describing GV came into being, which are of historical importance. These include the Iwase and Arakawa classifications, the Japanese Society for Portal Hypertension (JSPH) modification of the Hashizume classification, and the Italian Endoscopic Classification. Another simple classification differentiates GV into primary and secondary, the latter occurring after band ligation and eradication of esophageal varices (Additional file 1: Table 1) [5, 7, 8].

### The relevance of collateral pathway anatomy in gastric varices

The GVs are generally described and therapeutic decisions made based on their location and relationship with esophageal varices. Understanding the complex GV system is important in deciding on therapeutic options beyond endoscopic interventions. In general, via hepatofugal pathways, GV drain into the systemic circulation through two types of collateral systems. These are the gastroesophageal system, between the left gastric vein and the azygous vein *and* the gastrophrenic system between the gastric veins in the posterosuperior gastric wall; and left inferior phrenic vein at the gastrophrenic ligament near the bare area of the stomach. In isolated splenic vein thrombosis, the collateral circulation pathways form in hepatopetal manner [8].

**Fig. 1:**
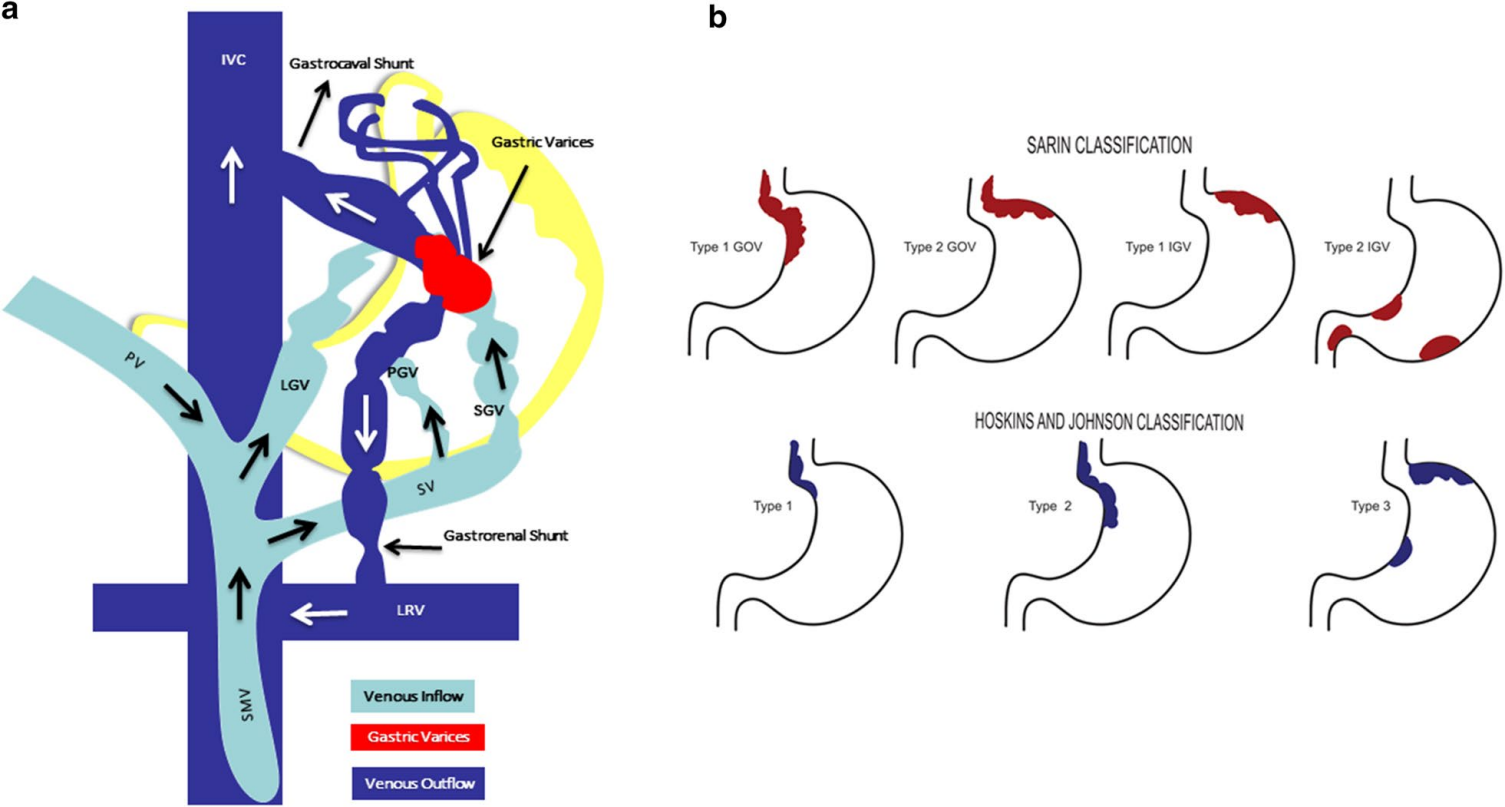
**a** The components of the gastric variceal system demonstrating the portal venous inflow, the gastric varix proper and the systemic venous outflow that together form the variceal complex; **b** endoscopic classification of the gastric varices, the Sarin and Hoskin and Johnsons’ systems. *IVC* inferior vena cava, *PV* portal vein, *LGV* left gastric vein, *SMV* superior mesenteric vein, *LRV* left renal vein, *SV* splenic vein, *PGV* posterior gastric vein, *SGV* short gastric veins. Illustrations used in this figure is created by listed authors (Cyriac Abby Philips and Sasidharan Rajesh)

The Type 1 GOV (of Sarin classification) drains through the esophageal and paraesophageal collateral veins; Type 2 GOV through inferior phrenic and esophageal veins; Type 1 IGV through left inferior phrenic vein and Type 2 IGV, in sinistral PH, through gastric veins. The afferent vein for Type 1 GOV is the anterior left gastric vein while for Type 2 GOV, it is the short gastric and posterior gastric veins, while in both, efferent are esophageal and paraesophageal veins. In IGV, the afferent is a gastric or splenorenal shunt and the inferior phrenic vein which terminate in the inferior vena cava [7, 12]. The GV also drains into the splenorenal shunt through the gonadal vein, or the gastrocaval shunt into the inferior vena cava through the inferior phrenic or pericardiophrenic vein. IGVs drain through hypertrophied inferior phrenic vein and left renal vein at the left adrenal vein in 85% [12, 13]. These detailed collateral and portosystemic shunt descriptions paved way for hemodynamic classifications that provided deeper anatomical insights on which interventional radiology-based management decisions for endoscopically difficult to control bleeding may be adeptly chosen, a cognizance lacking in the original, standard classification systems (Fig. 1).

### Cross-sectional imaging-based evaluation of the gastric variceal complex

Spontaneous portosystemic shunts (SPSS) are large collaterals that develop between the portal and systemic venous circulation that hypertrophy and enlarge to accommodate high blood volume and flow with increasing severity of PH. These can be divided into left and right-sided or central shunts. Left-sided shunts are those that are present to the left of midline or the left of the splenic confluence and mesenteric veins. The most common left-sided shunt is the gastrorenal shunt, which is present in 10% of patients with PH but is notable in 85% with GVs [14, 15].

The gastric variceal system consists of the gastrorenal shunt, the central part that is gastric varix proper, and the associated afferent portal venous collateral feeder vessels. The variceal complex consists of the afferent limb (portal inflow), a central portion (varix proper), and an efferent limb (systemic outflow). The portal inflow feeder vessels do not directly communicate with gastric varix proper and take part in the formation of varices outside the gastric wall, called para/extra-gastric or false GV. True gastric varix is the intragastric submucosal portion that bleeds into the lumen. Intragastric and the para-gastric varices together form the central portion of the gastric variceal complex. The extra-gastric and intragastric components may communicate with each other through a single or multiple perforator vein(s). The dominance anatomy of the portal inflow vessels is of great importance. In some patients, the dominant afferent vessel is the coronary or left gastric vein, while in others, it is the posterior gastric vein. In a highly complex gastric variceal system, triple dominance can be noted with multiple feeder systems (afferent limbs). When the short gastric veins become dominant afferent vessels in GV formation (usually in splenic or PV thrombosis), the variceal complex extends over the fundus, body, cardia, antrum and gastric outlets. This corresponds to the ‘diffuse-type’ of GV as per Iwase and Arakawa classification and which is absent from the Sarin classification [15, 16]. Verma and colleagues recently reported on the twenty-year experience of diagnosis and treatment of GV at a large tertiary university in which the authors described Type 3 GOV (esophageal varices with gastric varices extending over body, antrum, and pylorus), in 10.5% of patients, previously described by Iwase and Arakawa [17] The efferent or outflow from the gastric varix proper can be as simple as a single gastrorenal shunt or may become complicated with multiple outflow channels due to the involvement of inferior phrenic or pericardiophrenic veins. As the severity of PH increases, the shunt flow increases, and the shunt grows and travels caudally and posteriorly, reaching the retroperitoneal and other regions sometimes undergoing duplication at the site of drainage. Understanding the complex anatomy of the GV system and associated hemodynamic classifications are significant in planning a multitude of managements for bleeding GV such as endoscopic cyanoacrylate therapy only or shunt occlusion with or without variceal embolization, endoscopic ultrasound-guided coiling or transjugular intrahepatic portosystemic shunt placement [7, 8, 14–16].

### Hemodynamic classification of gastric varices

**Fig. 2:**
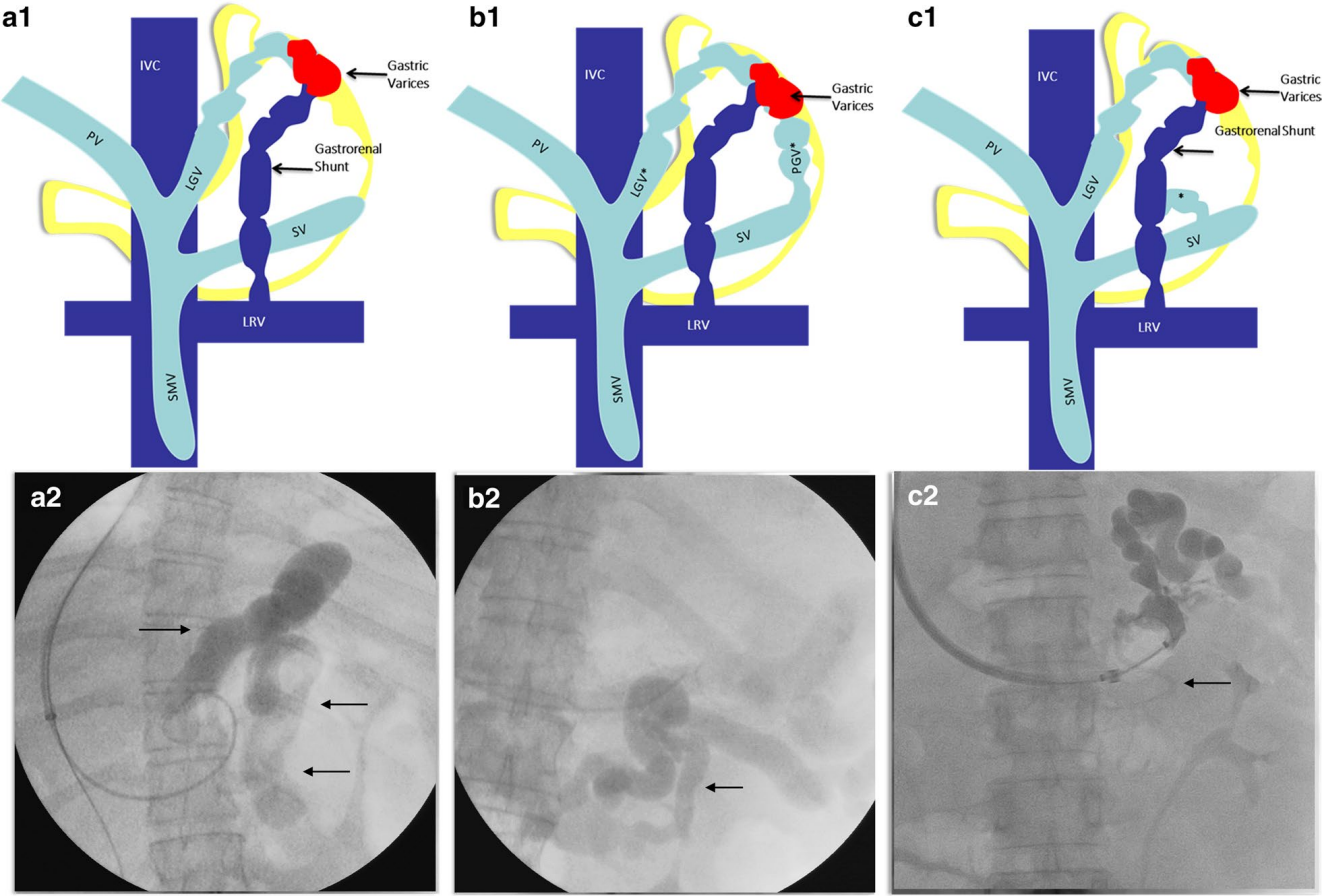
Classification of gastric varices according to afferent venous inflow—type 1 gastric varices are supplied by a single afferent gastric vein (**a1**, **a2**); type 2 gastric varices by multiple afferent gastric veins (**b1, b2**); and type 3 (**c1, c2**) are supplied by single or multiple gastric veins with co-existent gastric veins that are directly contiguous with a shunt, but without contribution toward gastric varices. *IVC* inferior vena cava, *PV* portal vein, *LGV* left gastric vein, *SMV* superior mesenteric vein, *LRV* left renal vein, *SV* splenic vein, *PGV* posterior gastric vein, *SGV* short gastric veins. Illustrations used in this figure is created by listed authors (Sasidharan Rajesh and Tom George)

**Fig. 3:**
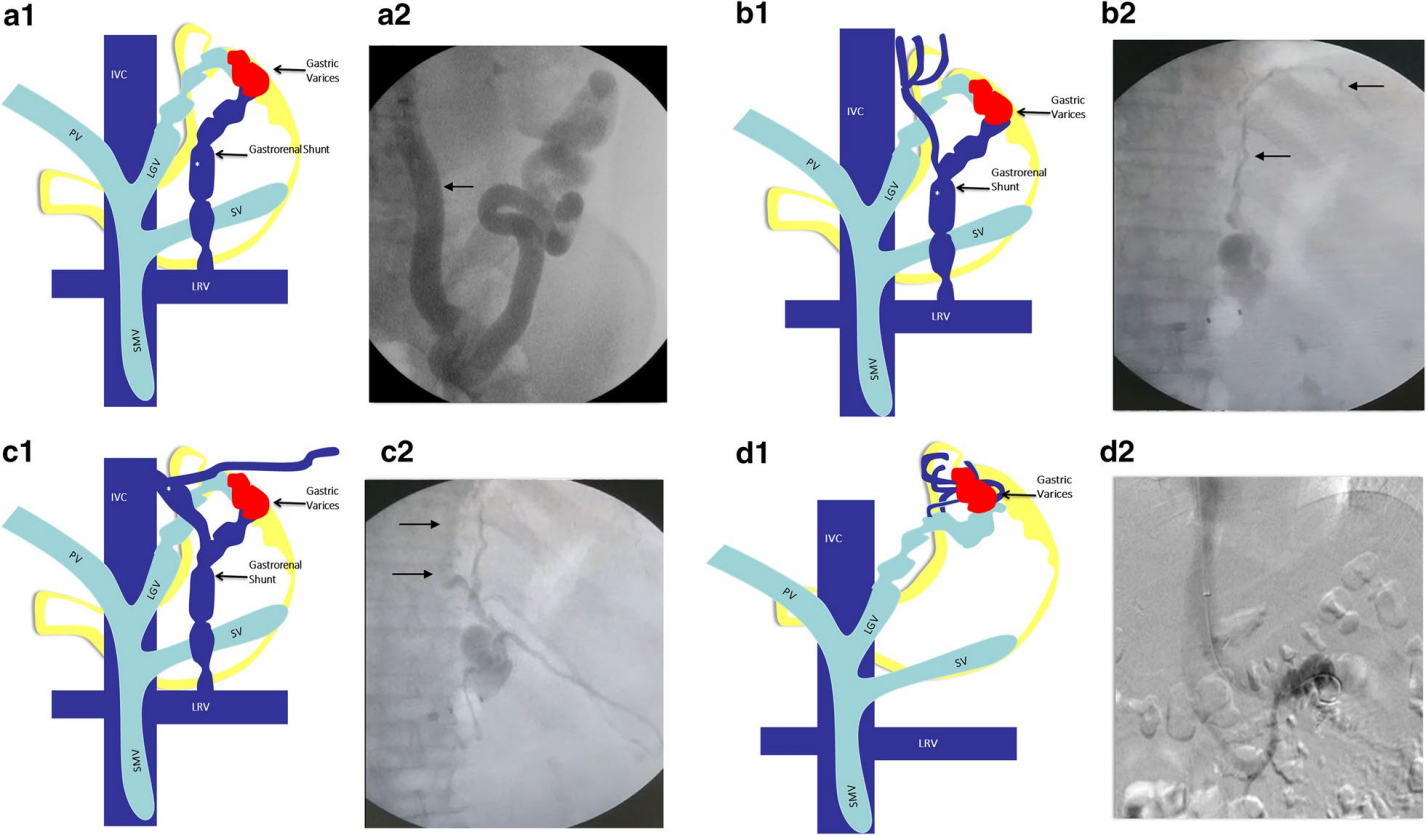
Classification according to efferent venous outflow—type A gastric varices (**a1, a2**) are contiguous with a single shunt, type B (**b1, b2**) with a single shunt and collateral veins, type C (**c1, c2**) with both gastrorenal and gastrocaval shunts and type D (**d1, d2**) are contiguous with multiple collaterals in the absence of shunt(s). *IVC* inferior vena cava, *PV* portal vein, *LGV* left gastric vein, *SMV* superior mesenteric vein, *LRV* left renal vein, *SV* splenic vein, *PGV* posterior gastric vein, *SGV* short gastric veins. Illustrations used in this figure is created by listed author (Sasidharan Rajesh)

The recent classification of GVs, is based on the hemodynamics of afferent and efferent flow rather than location and extent. These classifications, related to afferent and efferent circulation, improve therapeutic options beyond conventional endoscopy-based treatments (Figs. 2, 3).

#### Based on afferent/inflow hemodynamics

Kiyosue classification divides GV into three types. In Type 1, a single afferent vein supplies varix; in Type 2, multiple afferent veins supply the GV and in Type 3, single or multiple afferent veins supply the GV through a shunt (indirectly). The commonest afferent vein in Type 1 is the left gastric vein or coronary vein and in Type 2, the left gastric vein and posterior gastric vein. In the Saad–Caldwell classification based on dominance, Type 1 GV are associated with a single afferent vein (left gastric vein); in Type 2, the afferent vein is the posterior gastric vein or the short gastric veins; in Type 3, equal dominance is noted between multiple afferent veins, and in Type 4, multiple afferent veins form in presence of splenic vein thrombosis (Table 1) [15, 16, 18].

**Table 1:** Hemodynamic classification of gastric varices based on portal outflow/efferent system

Classification system	Clinical relevance
*Kiyosue classification*	In Type A, shunt occlusion as the treatment of modality would suffice to control variceal bleeding not controlled with endoscopic therapy. In type B, feasibility of shunt occlusion might be less and hence transjugular intrahepatic portosystemic shunt placement is a better option to obliterate all of the collateral pathways In type C, transjugular intrahepatic portosystemic shunt placement along with shunt emobilization of large portosystemic shunts could be the best option in ideal candidates In Type D, in the presence of endoscopic failure, transjugular intrahepatic portosystemic shunt placement could become the best option
Type A: single draining shunt Type B: single shunt and multiple collateral veins B1: small collateral veins B2: medium sized collateral B3: large collateral veins with high flow without shunt Type C: more than one shunt present C1: small sized second shunt that cannot be catheterized C2: presence of second shunt large enough to be catheterized Type D: shunt is not present and the varices drain through small collaterals	
*Saad–Caldwell classification*	In Type D, embolization procedures may not suffice to prevent rebleeding or control active bleeding due to the complex anatomy, and hence, transjugular intrahepatic portosystemic shunt placement could become the best option for prevention of further bleeding
Type A: single draining shunt Type B: single shunt and multiple collateral veins B1: small collateral veins B2: medium sized collateral B3: large collateral veins with high flow without shunt Type C: more than one shunt present C1: small sized second shunt that cannot be catheterized C2: presence of second shunt large enough to be catheterized Type D: shunt is not present and the varices drain through small collaterals D1: predominance of systemic vein drainage is not obvious and any vein, out of inferior phrenic, hemiazygos tributaries, and intercostals veins or adrenal veins may be predominant D2: morphology similar to D1, but predominant systemic venous draining vein is usually 4.3 mm in diameter through unconventional systemic veins	
*Hirota—BORV classification*	In Type A, shunt embolization can help obliterate gastric varices In Type B, transjugular intrahepatic portosystemic shunt placement with or without shunt embolization can help obliterate varices In Type C, transjugular intrahepatic portosystemic shunt placement and shunt embolization need to be performed for large shunts for complete variceal disease management In Type E, an antegrade approach for shunt embolization is more feasible than a retrograde approach since balloon sizes may not be available and the shunt flow is high
Type A: single draining shunt Type B: single shunt and multiple collateral veins B1: small collateral veins B2: medium sized collateral B3: large collateral veins with high flow without shunt Type C: more than one shunt present C1: small sized second shunt that cannot be catheterized C2: presence of second shunt large enough to be catheterized Type D: shunt is not present and the varices drain through small collaterals Type E: gastrorenal shunt too large for balloon occlusion procedures	

#### Based on efferent/outflow hemodynamics

In the Kiyosue classification of the gastric variceal system based on the outflow, four types are described. In Type A, the GVs are associated with a single draining shunt, most commonly the gastrorenal shunt. In Type B, drainage occurs through the gastrorenal shunt and associated multiple collateral veins. Type C GVs are associated with multiple shunts without additional collaterals. In Type D, multiple collateral veins are present without large shunts. In the Hirota-BORV classification, the descriptions are similar to Kiyosue (Type A–D) but with the addition of Type E, in which the gastrorenal shunt is too large for transvenous retrograde balloon occlusion. In such situation, an antegrade approach is more feasible for shunt and variceal embolization (Table 2) [15, 16].

**Table 2:** Hemodynamic classification of gastric varices based on balloon occluded transvenography

Classification system	Clinical relevance
*Hirota classification*	Only endoscopic guided or endoscopic ultrasound guided therapy may help in obliteration of varices of Type 1 and 2 Transjugular intrahepatic portosystemic shunt placement is ideal for Type 3 and 4 related bleeding Transjugular intrahepatic portosystemic shunt placement and shunt embolization is ideal in Type 5
Grade 1: gastric varices well opacified without any collateral vein evidence Grade 2: contrast opacification in gastric varices for ≥ 3 min in the presence of small and few collateral veins Grade 3: contrast opacification of gastric varices partial and disappears within 3 min with medium to large collateral veins which were few in number Grade 4: non-contrast opacification of gastric varices and presence of many large collaterals Grade 5: shunt cannot be occluded because of very large size of shunt and rapid blood flow	
*Fukuda classification*	Based on hemodynamic features involving the superior mesenteric and celiac angiography findings In Type 2 and Type 3 with left gastric vein dominance, rebleeding can be noted with only endoscopic management and hence transjugular intrahepatic portosystemic shunt placement may become the treatment of choice In those associated with shunts, shunt embolization with or without transjugular intrahepatic portosystemic shunt placement may be superior to only endoscopic therapy
Type 1: left gastric vein dominant gastric variceal complex Type 2: separation between the esophageal varices (left gastric vein dominant) and the gastric varices (posterior gastric vein/superior gastric vein dominant) Type 3: highly complex system consisting of both right and left sided feeding vessels Type 4: right sided dominance only of gastric variceal system	
*Matsumoto classification*	Classification system for gastric varices for predicting the aggravation of esophageal varices after balloon occluded retrograde transvenous occlusion procedure Based on left gastric angiography Aggravation of esophageal varices grade occurs in Type 1B varices
Type 1: portosystemic flow in the gastrorenal shunt A: hepatopetal flow B: hepatofugal flow Type 2: no portosystemic flow in the gastrorenal shunt A: hepatopetal flow B: hepatofugal flow	

#### Based on balloon occluded retrograde transvenography

**Fig. 4:**
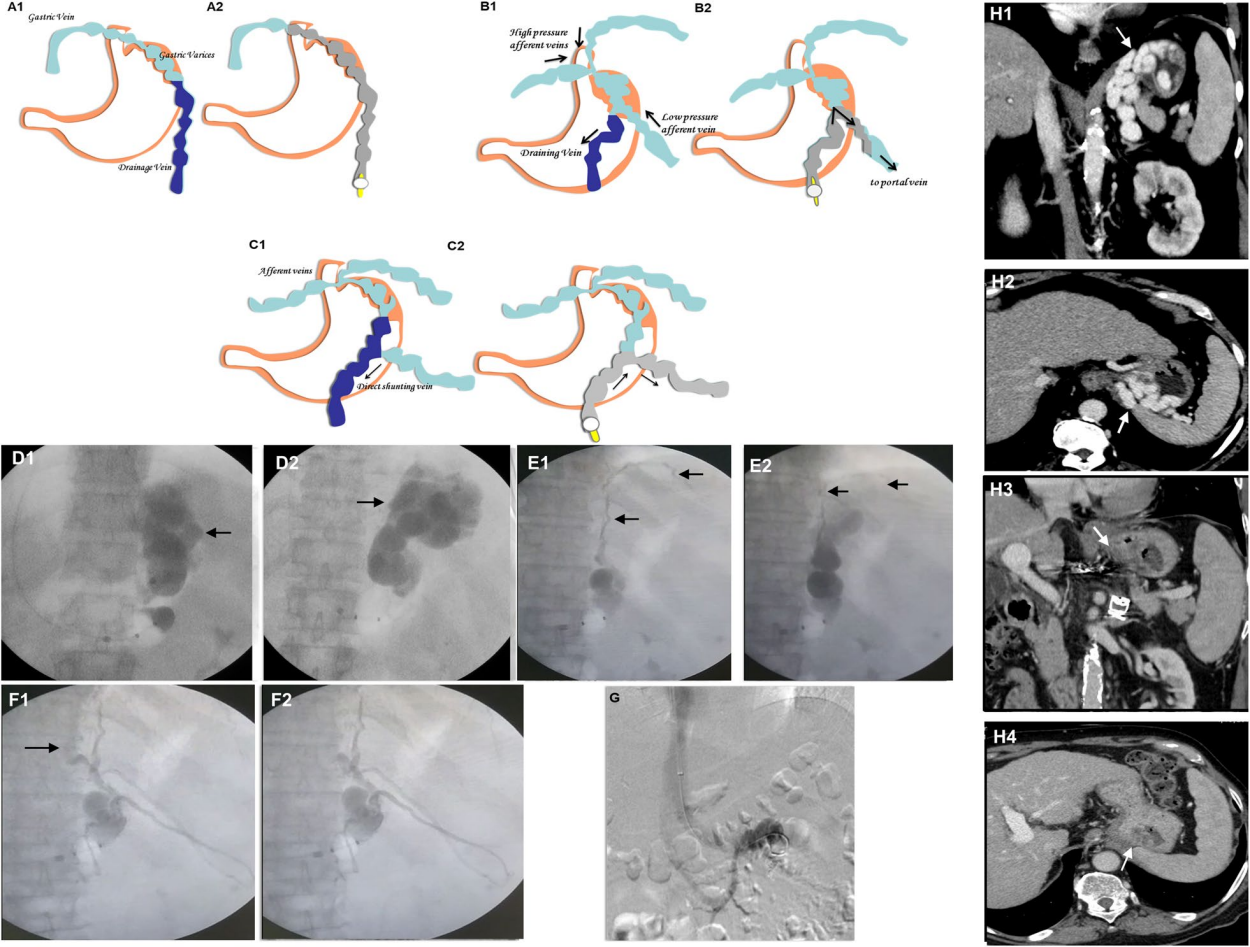
Clinical significance of afferent venous inflow (**a**–**c**) and outflow (**d**–**g**) of gastric varices during shunt embolization procedure. In type 1 gastric varices with ideal anatomy for occlusion, post sclerosant injection varices fully fill and are completely obliterated (**a1, a2**); in type 2 varices, with multiple afferents, the sclerosant tends to flow toward low-pressure gastric collateral increasing risk portal vein thrombosis [(**b1, b2** (arrows)]; in type 3, the sclerosant tends to flow in the direction of large shunt [(**c1, c2** (arrows)]; in type A (**d1, d2**), sclerosant completely fills the varices without run-off; in type B the sclerosant flows into the systemic veins (**e1**, arrows) and hence the associated high flow collateral vein needs additional gelfoam occlusion (**e2**, arrows) before sclerosant injection; in type C in presence of both gastrocaval (**f1**, arrow) and gastrorenal shunts the sclerosant tends to flow into a systemic vein through the second shunt. Hence the outflow shunt is occluded first with gelfoam (**f2**, arrow); in type D gastric varices (**g**), without draining veins, transjugular intrahepatic portosystemic shunt placement is ideal choice for complete variceal complex obliteration; **h** classical pre (**h1, h2**) and post (**h3, h4**) computed tomography demonstration of obliteration of gastric varices associated with a single large efferent shunt. Illustrations used in this figure is created by listed author (Sasidharan Rajesh)

Hirota classification is specifically based on real-time features of angiographic opacification of gastric varices (from grade 1–5). In Grade 1, GVs are well opacified without evidence of collateral circulation, while in Grade 5, the opacification of varices occurs minimally due to the presence of large shunt and rapid volume run-off. In the Fukuda classification, Type 1 includes GVs associated with the dominant left gastric vein, while in Type 2, the left gastric vein supplies the esophageal component of the variceal complex while the posterior or short gastric veins supply the gastric component. Type 3 include both left and right feeder vein dominant gastric variceal complex while Type 4 is associated with purely right-sided dominant supply. Matsumoto and colleagues classified GVs based on predicted aggravation of esophageal varices after embolization procedures. In Matsumoto Type 1 there is associated portosystemic flow in the gastrorenal shunt, and Type 2 portosystemic shunt flow is absent. In both, subtype A is associated with hepatopetal flow, while subtype B is associated with hepatofugal flow in the left gastric vein. Worsening of esophageal varices is associated with Matsumoto Type 1B in which after shunt embolization, backward flow into the left gastric vein results in increasing grades of esophageal varices (Additional file 1: Table 2) [18, 19]. Clinical significance of hemodynamics (inflow and outflow) based classification and associated treatment of GVs during shunt occlusion procedures, beyond endoscopic management is shown in Fig. 4.

## Treatment

### Primary prophylaxis of gastric variceal bleeding

In patients with GVs who have not bled, similar to the prevention of acute variceal bleeding from esophageal varices, the use of nonselective beta-blockers has been suggested. The role of endoscopic cyanoacrylate glue injection and endoscopic band ligation (EBL) as options for primary prophylaxis in gastroesophageal varices remain unclear. In a study conducted from a single center in India, endoscopic glue injection was found to be associated with lower bleeding and mortality compared to nonselective beta-blockers [20]. Kang et al. demonstrated the long-term efficacy of prophylactic cyanoacrylate glue therapy in 27 patients with high-risk GVs with 6-months cumulative survival of 75% [21]. The Baveno VI consensus and American Association for the Study of Liver Diseases recommend the use of non-selective beta-blockers [22].

Bhat and colleagues studied the primary prophylaxis of gastric variceal bleeding using EUS guided glue injection and found that only 5% bled at 449 days follow up. Further studies on EUS based therapy for prevention of bleeding in GV are lacking [23]. In the study by Koziel et al. on EUS-guided obliteration of GVs using vascular coils only or coils with CYA injections for primary and secondary prophylaxis for GV haemorrhage, technical success was 94% without serious complications [24]. Nonetheless, this was a small series with retrospective methodology and inherent bias. Primary TIPS is not recommended for prevention of GV bleeding. Balloon-retrograde transvenous occlusion (BRTO) and it's variant techniques such as coil-assisted retrograde transvenous occlusion (CARTO), plug-assisted retrograde transvenous occlusion (PARTO), balloon antegrade transvenous occlusion (BATO) and our group described novel techniques such as the ‘direct’ (D)-PARTO or direct coil-assisted antegrade transvenous occlusion (CAATO) are not evaluated in high-quality randomized trials for prevention of first gastric variceal bleeding and hence cannot be recommended as primary prophylaxis [25].

### Management of acute gastric variceal bleeding and secondary prophylaxis

On diagnostic endoscopy, gastric variceal bleeding is confirmed in the presence of active bleeding from a visualized varix, presence of adherent clot or stigmata of recent haemorrhage over the GV and recurrent bleeding in a patient with PH and presence of GV in the absence of other identifiable sources of bleeding [26].

The general measures for initial optimization of clinical status to prevent further deterioration due to acute gastric variceal bleeding are similar to those followed in esophageal variceal bleeding. This includes airway protection through endotracheal intubation to prevent aspiration, maintaining minimum systolic blood pressure of 70 mm Hg for performing urgent diagnostic and therapeutic endoscopy and the judicious use of packed red cells for target hemoglobin levels between 7 and 8 g/dL (21% haematocrit). Volume expansion and coagulation correction using fresh frozen plasma or plasma expanders lead to severe adverse clinical events in patients with cirrhosis and variceal bleeding and must be avoided. A conventional dose of two fresh frozen plasma units can only replace 10% of the clotting factors. Large volume coagulation correction can lead to worsening PH, sepsis, sinister systemic immunomodulation, and rebleeding. In cirrhosis, a minimum platelet count 56,000/mL corresponds to adequate thrombin generation and is the ideal target for endoscopic interventions. Similarly, maintaining fibrinogen level > 120 mg/dL also improves haemostatic effects [27–29]. Although the use of vasoactive agents for the reduction in portal pressure and control of rebleeding specific to gastric variceal bleeding is unavailable in literature, the same line of supportive therapy as for esophageal variceal bleeding, is currently recommended. Wang et al. in their systematic review and meta-analysis showed that there was no difference between vasopressin/terlipressin and somatostatin/octreotide in the prevention of re-bleeding after the initial treatment of bleeding esophageal varices [30]. Antibiotic prophylaxis, lactulose for the prevention of hepatic encephalopathy along with other supportive measures that include varying degrees of organ support depending on the severity of systemic dysfunction is mandated in GV bleed [31]. In a patient with active bleeding that preclude endoscopic treatment, temporizing measures such as intragastric balloon tamponade can be utilized. These devices can only be placed for a maximum of 24 h within which definitive treatment has to be carried out. Given its large volume capacity, a Linton-Nachlas tube is considered ideal for gastric variceal bleeding [32].

### Endoscopic band ligation

Endoscopic band ligation (EBL) is the initial treatment of choice in the management of acute esophageal variceal bleeding. Initially, several small patient series demonstrated that EBL was safe and effective for bleeding GV. Two randomized controlled trials comparing EBL to cyanoacrylate glue therapy showed that initial haemostasis was lower and rebleeding rates higher (63% and 72% at 2 and 3 years respectively) in the former. In the absence of cyanoacrylate glue, EBL can be considered in patients with Type 1 GOV bleeding for initial control of bleeding until further definitive management can be undertaken [33, 34].

### Sclerotherapy

Injection sclerotherapy for GV has been demonstrated to be less effective than what is noted with esophageal varices. The agents used for sclerotherapy include ethanolamine oleate, sodium tetradecyl, glucose solutions, and acetic acid. High blood flow within the GV results in the early flush of injected sclerosants, reducing its efficacy. In such situations, larger volumes of injection can be contemplated. However, in reality, it leads to adverse events such as febrile illness, severe retrosternal discomfort, ulcerations, mediastinitis, embolization in the presence of large portosystemic shunts and perforations that can result in approximately 50% mortality. The rebleeding rates with sclerotherapy alone can be as high as 90%, of which 50% bleeds are secondary to injection site ulcerations. Sclerotherapy has greater success for control of bleeding and prevention of rebleeding in esophageal variceal disease [35, 36]. Currently, EBL or cyanoacrylate glue injection is considered the treatment of choice for Type 1 GOV bleeding and cyanoacrylate glue injection for Type 2 GOV and isolated GV. Some authors have used EBL along with sclerotherapy for management of Type 1 GOV bleeding with an injection of 1 mL of sclerosant above the site intended for band ligation. The success rate for haemostasis with this approach is close to 90% with the risk of rebleeding in 33%. EBL should only be performed in patients with bleeding from small Type 1 GOV in which both the mucosal and contralateral wall of the vessel undergoes complete suction into the ligator, without which the likelihood of band detachment is high leading to ulceration of the overlying vessel and catastrophic secondary bleeding [35, 36].

### Endoscopic cyanoacrylate glue therapy

N-butyl-2-cyanoacrylate (NBC) is a monomeric tissue adhesive that rapidly polymerize on contact with blood leading to hardening of varix, cast formation, and obturation. The NBC is the most commonly employed agent for glue therapy and undergoes polymerization within 20 s of contact with blood inside the variceal lumen. Lipiodol (ethiodized oil composed of iodine combined with ethyl esters of fatty acids of poppyseed oil, primarily as ethyl monoiodostearate and ethyl di-iodostearate) or normal saline is sometimes used to avoid occlusion in the endoscopy channel. A 1:1 mixture is usually recommended and can reduce the risk of embolization. It is recommended that a 3.7 mm width channel endoscope be utilized for ease in glue administration. Some newer glue products such as 2-octyl-cyanoacrylate and NBC mixed with methacryloyloxy-sulfolane do not require dilutional agents due to the slow polymerization time [37, 38]. In a Cochrane Database Review, a meta-analysis of three randomized controlled trials comparing cyanoacrylate glue therapy versus EBL demonstrated both therapies to be effective for control of bleeding, but significantly lower rates of rebleeding was noted with the former. These studies included mostly Type 1 GOV bleeds and utilized NBC [39].

### Endoscopic thrombin injection and inorganic haemostatic powder spray

Another treatment modality infrequently used in gastric variceal bleed control is thrombin injection in which catalysation of fibrinogen to fibrin with additional platelet function augmentation enhances cot formation within the bleeding varix. This is an attractive alternative to glue therapy where expertise is unavailable and has fewer side effects and systemic complications. Five millilitres of thrombin have the potency to coagulate one litre of blood in less than a minute. Even though thrombin treatment was found beneficial in controlling bleeding from GV, especially Type 2 GOV in small single-center series, high-quality studies were lacking [40–43]. Recently, Lo and colleagues, in a prospective randomized trial, showed that endoscopic thrombin injection was similar to glue injection in achieving successful haemostasis of acute GV bleeding but with higher incidence of complications associated with the latter [44]. Few reports demonstrating the use of inorganic absorbent powder TC-325 haemostatic spray (Hemospray®, Cook Medical, IN, USA) in patients with refractory gastric variceal bleeding after the failure of glue injection therapy has been published in the literature. The issue with haemostatic powder spray is that it can act only in the presence of active bleeding during endoscopy [45].

### Endoscopic-ultrasound guided therapy for gastric varices

**Fig. 5:**
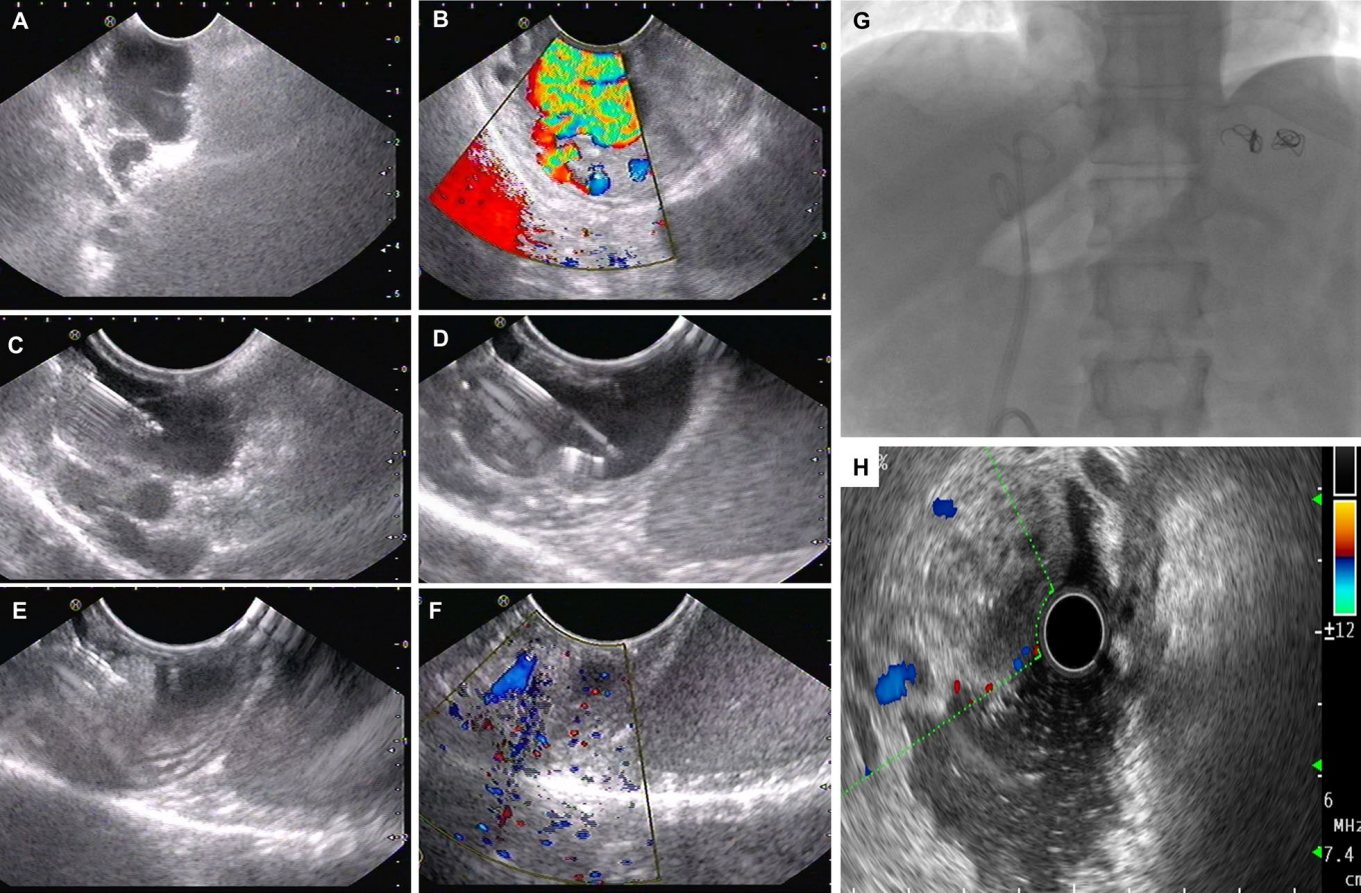
Endoscopic ultrasound (EUS) guided coiling and glue therapy for recurrent bleeding from gastric varices. Large gastric varix is noted (**a**) with turbulent blood flow within (**b**). A 22 G aspiration needle is pushed into the varix (**c**) and coils inserted into the varix lumen under direct vision (**d**). Additionally, 1.5 mL of cyanoacrylate glue is injected to completely obliterate the variceal lumen (**e**). Immediate post treatment, the turbulent flow has almost disappeared from variceal complex (**f**). Six months after treatment, the coils are visible on abdominal fluoroscopy (**g**) and there is complete obliteration with loss of Doppler signals from the varix on repeat EUS (**h**)

EUS color Doppler can help distinguish GV from gastrointestinal tumors and prominent gastric folds and allows real-time confirmation of GV obliteration through precise identification of perforating feeder vessels and accurate delivery of tissue adhesive decreasing the amount of glue injected and reducing the risk of embolization (Fig. 5). Romero-Castro et al. performed a proof-of-concept study on EUS-guided glue therapy for bleeding GV and utilized lipiodol to localize feeder vessels before glue use accurately [46]. Lee et al. showed that late rebleeding rate beyond 48 h was significantly lower in patients with GV bleeding receiving EUS guided glue injections every 2-weeks until eradication [47]. In another single center study, 90% of patients experienced complete haemostasis after glue injection into the afferent vessels confirmed on color Doppler, without rebleeding events in the short term [48].

EUS-guided coiling of GV was shown to enhance haemostasis in multiple series. The metal coils, made from synthetic stainless-steel fibre induce clot formation and thrombosis of varix. Usually, a 19-G access needle is utilized, but 22-G needles for the deployment of smaller coils are also available. Levy and colleagues were the first to report on EUS-guided coiling of ectopic GV. A multicenter cohort study by Romero-Castro and colleagues demonstrated that there were no significant differences between glue injection compared to coiling for haemostasis of bleeding GV at 180-days. Nevertheless, the mean endoscopic session time and the number of sessions required for variceal obturation was lower in patients receiving EUS-guided coiling [49, 50]. The combined use of EUS-guided coil placement along with cyanoacrylate glue injection results in *en-mass* ‘scaffold’ formation which is associated with very efficient control of bleeding and reduction in the rate of rebleeding. Combined EUS-guided therapy promoted gastric variceal eradication in 96% of treated patients with a single sitting with only 16% experiencing rebleeding over a follow-up period of 6-months without any minor or major adverse events [51]. Similar findings were demonstrated by Bhat et al. in their study on 100 patients. However, adverse events in the form of pulmonary embolism and self-limited abdominal pain occurred in 5% [52]. A recently performed systematic review and meta-analysis showed that EUS combination therapy with coil embolization and glue injection was a preferred strategy for the treatment of GV over EUS-based monotherapy [53].

#### Endovascular therapy for bleeding gastric varices

##### Transjugular intrahepatic portosystemic shunt (TIPS)

The role of TIPS in controlling acute variceal bleeding in the event of rebleeding or uncontrolled bleeding from esophageal varices is well documented. Even though TIPS can promote haemostasis in acute GV bleeding, varices can persist and bleed at lower portal pressures than esophageal varices. Previous retrospective studies have shown that in patients with GV haemorrhage undergoing TIPS placement in the absence of adjuvant variceal embolotherapy, the GV remained patent in 65% with rebleeding in 27% and 90-days mortality of 15%. Another study also reported that 50% of patients post TIPS had persistence of GV with 27% rebleed rates [54, 55]. A meta-analysis comparing TIPS to endoscopic variceal sclerotherapy (EVS) in the management of GV bleeding in terms rebleeding, hepatic encephalopathy and survival demonstrated improved benefits of TIPS in the prevention of GV rebleeding that was associated with an increased risk of encephalopathy with comparable survival between study groups [56].

Various theories have contemplated the ineffectiveness of TIPS alone for complete control of bleeding from GV. The ‘proximity’ theory states that esophageal varices are well decompressed after TIPS since the left gastric vein supplying the varices are small and close enough to benefit from decompression through shunt creation. The GV, on the other hand, is farther away, larger, and associated with multiple afferents depending on the collateral anatomy of the variceal complex. As per the ‘throughput’ theory, low-pressure shunts from large-calibre inflow and outflow vessels associated with GV compete with and effectively decompress the TIPS stent leading to the persistence of varices. The ‘recruitment’ theory states that new afferent vessels form after treatment of a gastric variceal system post TIPS due to the complexity in afferent and efferent flow pathways, all of which do not undergo decompression or undergo only partial embolization unexpectedly. In such situations, shunt occlusion and TIPS may be more effective than TIPS alone [57, 58].

##### Retrograde or antegrade transvenous embolization of gastric varices

The American College of Radiology Appropriateness Criteria Committee on interventional radiology recently recognized BRTO as an alternative to TIPS in specific clinical situations for treatment of GV. As per current conservative practice, BRTO is reserved for those patients who are ineligible for TIPS. However, with improvement in understanding of hemodynamic physiology associated with the variceal disease, this has changed to incorporate a combination of endovascular therapies. A meta-analysis on post-procedure outcomes in 1016 patients who underwent BRTO for management of bleeding GV demonstrated technical success, i.e., complete thrombosis of the GV on short-term follow up imaging and control of active bleeding among 96.4% patients. Absence of rebleeding and no bleeding in high-risk GV was notable in 97.3% on follow up. However, most studies were retrospective in nature and included patients who underwent primary prophylactic BRTO for high-risk GV [59]. In another meta-analysis, on clinical outcomes in GV bleeding among 353 patients undergoing TIPS (n = 143) or BRTO (n = 210), it was found that no significant differences were notable with respect to technical success, haemostasis and complication rates between both treatments. Nevertheless, rebleeding and hepatic encephalopathy were significantly lesser in those who underwent BRTO [60]. Adverse events associated with conventional BRTO include fever, chest pain, gastrointestinal symptoms, haemoglobinuria, ascites, and pleural effusion. It was shown that the occlusion of a large gastrorenal shunt could increase the hepatic venous pressure gradient by up to 44% from the baseline. BRTO was found to aggravate pre-existing esophageal varices (ranging from 30 to 68%), leading to variceal bleeding even though associated death is never reported. In this context, a pre-shunt-occlusion endoscopy and prophylactic band ligation of large or high-risk esophageal varices are prudent.

**Fig. 6:**
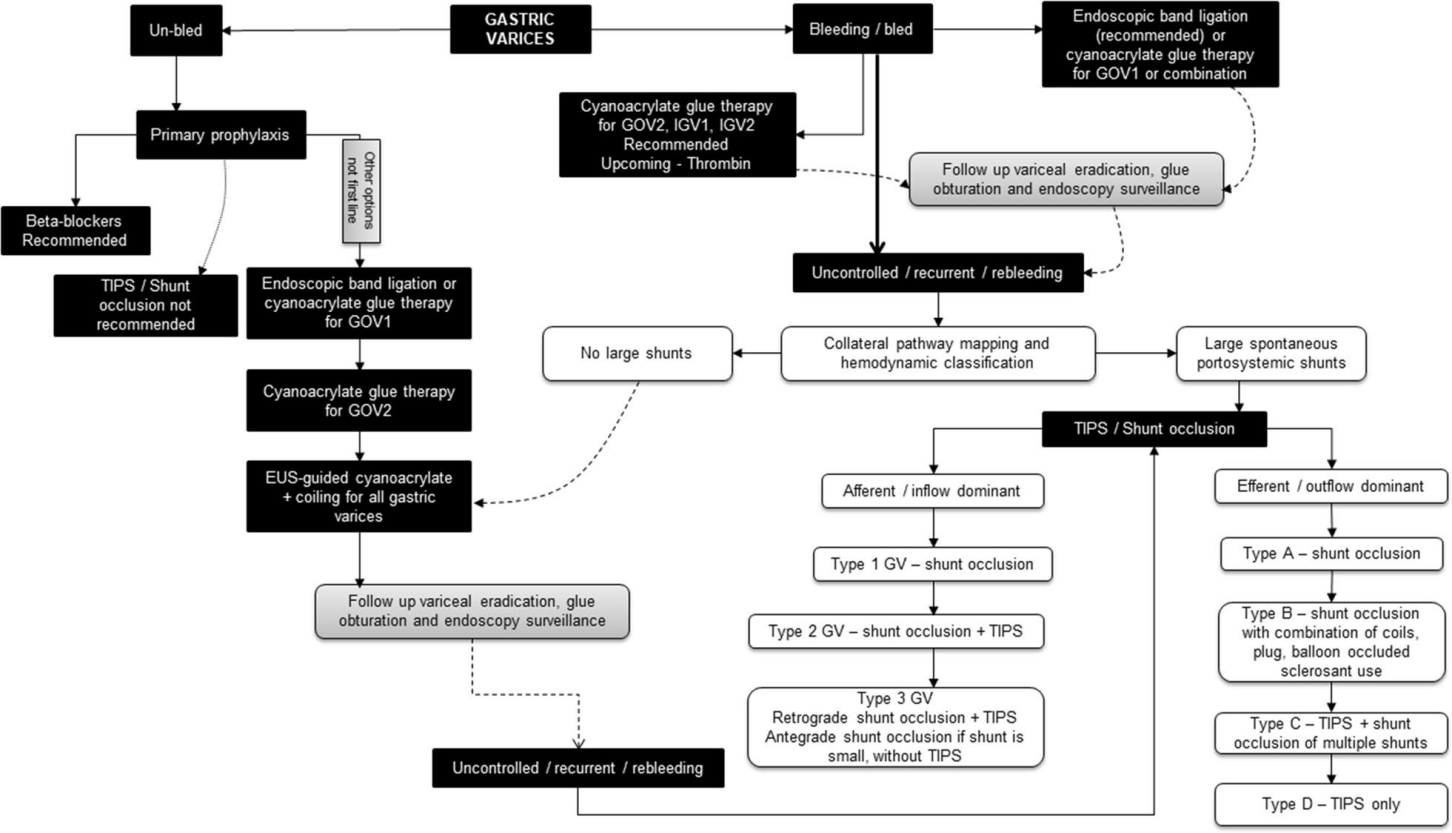
Updated treatment algorithm for gastric varices. *GOV* gastroesophageal varices, *IGV* isolated gastric varices, *TIPS* transjugular intrahepatic portosystemic shunt

In some patients, with GV bleeding, the combination of endovascular procedures could be more efficacious than singular treatments which in turn depends on the variceal collateral pathway anatomy. For example, as per the afferent flow classification, patients with Kiyosue type 1 GV can be easily managed with only shunt embolization. In contrast, TIPS placement would benefit those patients with GV and associated multiple collateral afferents in the absence of a dominant shunt (Type 2 of Kiyosue classification). Alternatively, in patients with afferent and efferent shunts as well as multiple collaterals (such as Kiyosue or Saad Caldwell Type C2), a combination of TIPS and shunt embolization could be more beneficial. Shunt embolization, along with TIPS placement, negates the high flow through the shunt, reduces rebleeding rates, improves TIPS efficacy, shunt patency and flow and decreases the incidence of hepatic encephalopathy [32, 58, 59]. In patients with large portosystemic shunts, it is not uncommon to notice an attenuated portal vein that is difficult to cannulate for the TIPS procedure. Shunt embolization improves portal vein inflow and increases portal vein diameter making a technically challenging TIPS procedure far more comfortable to perform. A combination of multiple embolization techniques, such as inflow modulation through coils or balloon occlusion followed by sclerosant injection and outflow modulation utilizing a plug, can lead to complete embolization of the variceal system with a reduction in sclerosant migration to untargeted regions. There are no published multicenter series of randomized trials on TIPS and combination shunt embolization procedures. Saad and colleagues reported outcomes in 36 patients undergoing BRTO procedure for gastric variceal bleeding in whom 9 underwent simultaneous TIPS placement. It was shown that the ascites and hydrothorax free rate for BRTO versus BRTO + TIPS at six months and one year was 58% and 29% compared to 100% and 100%, respectively. A significant reduction in recurrence of haemorrhage was also noted in the combination group demonstrating the fact that TIPS improved the PH burden developing after BRTO. Another prospective randomized controlled trial of TIPS alone versus TIPS with adjunctive left gastric vein embolization found a significant reduction in 180 days overall rebleeding rate in the embolization group [61, 62]. In a meta-analysis that compared the incidence of shunt dysfunction, variceal rebleeding, encephalopathy, and death between patients treated with TIPS alone and those treated with combined variceal embolization it was shown that variceal embolization during TIPS procedure improved the prevention of rebleeding, but no significant differences were identified concerning shunt dysfunction, encephalopathy, or mortality [63]. Thus, the treatment of gastric variceal bleeding has evolved through the years and is currently far from the current standard recommendations to better suit the patient, dependent on the hemodynamic classification and with reasonable control of portal hypertensive complications. An algorithm for treatment decisions regarding gastric variceal bleeding is shown in Fig. 6.

### Conclusion

Gastric variceal haemorrhage is associated with high rebleeding rates and mortality than esophageal variceal bleeding. Endoscopic cyanoacrylate glue therapy is the current standard recommendation for the management of gastric variceal bleeding. However, with a better understanding of the anatomic and hemodynamic components associated with the gastric variceal system, advanced options for bettering clinical outcomes are in evolution. These include EUS assisted combination approaches and multiple endovascular techniques including TIPS and shunt embolization or their combinations that can be offered to patients, depending on the underlying liver disease severity, collateral pathway anatomy, affordability and availability of technical expertise.

## Supplementary information

**Additional file 1: Supplementary Table 1**. Classification systems of gastric varices. **Supplementary Table 2**. Hemodynamic classification of gastric varices based on portal inflow/afferent system.

## Data Availability

On request from the corresponding author, Cyriac Abby Philips.

## Abbreviations

TIPS: Transjugular intrahepatic portosystemic shunt
GV: Gastric varices
GOV: Gastroesophageal varices
IGV: Isolated gastric varices
HVPG: Hepatic venous pressure gradient
EUS: Endoscopic ultrasound
JSPH: Japanese society for portal hypertension
SPSS: Spontaneous portosystemic shunt
EBL: Endoscopic band ligation
NBC: N-butyl cyanoacrylate
BRTO: Balloon-occluded retrograde transvenous occlusion
CARTO: Coil assisted retrograde transvenous occlusion
PARTO: Plug assisted retrograde transvenous occlusion
BATO: Balloon antegrade transvenous occlusion
D-PARTO: Direct PARTO
CAATO: Coil assisted antegrade transvenous occlusion
