# Supplementary material for: Beyond the scope and the glue: update on evaluation and management of gastric varices
Source: BMC Gastroenterol. 2020 Oct 30;20:361. doi: 10.1186/s12876-020-01513-7 (PMC7602314; doi:10.1186/s12876-020-01513-7)
Supplement: Supplementary file 1 — Additional file 1: Supplementary Table 1. Classification systems of gastric varices. Supplementary Table 2. Hemodynamic classification of gastric varices based on portal inflow/afferent system. [file 12876_2020_1513_MOESM1_ESM.docx]

**Supplementary table 1: Classification systems of gastric varices**

| **CHOI CLASSIFICATION** | **HOSKINS AND JOHNSON CLASSIFICATION** |
| --- | --- |
| F1 – mild gastric varices  F2 – moderate gastric varices  F3 – severe gastric varices | Type 1 – inferior extension of oesophageal varices across the squamocolumnar junction  Type 2 – gastric varices located in fundus, which appear to converge to cardia with oesophageal varices  Type 3 – gastric varices in fundus or body in absence of oesophageal varices |
| **MATHUR’S CLASSIFICATION** | **HASHIZUME CLASSIFICATION** |
| Type 1 – esophageal varices with lesser curvature varices  Type 2 – esophageal varices with fundal varices  (2a – subcardiac and 2b – diffuse fundal)  Type 3 – isolated fundal varix  (3a – secondary to splenic vein thrombosis, 3b – secondary to generalized portal hypertension)  Type 4 – lesser curvature gastric varices with oesophageal varices with fundal varices  Type 5 – antral varices | Form – tortuous (F1), nodular (F2) or tumorous (F3)  Location – anterior (La), posterior (Lp), lesser (Ll) and greater curvature cardiac (L) or fundic areas (Lf)  Colour – red (Cr) or colour white (Cw) and thin-walled focal redness on the varix as red colour spot (RCS) |
| **SARIN CLASSIFICATION** | **IWASE CLASSIFICATION** |
| Gastro-oesophageal varices Type 1 – continuation of oesophageal varices into the lesser curvature (GOV1)  Gastro-oesophageal varices Type 2 – esophageal and fundal varices are present in continuity with the greater curvature (GOV2)  Isolated gastric varices Type 1 – fundal varices are present in the cardia in the absence of oesophageal varices (IGV1)  Isolated gastric varices Type 2 – fundal varices present in the stomach outside of cardio-fundal region or first part of duodenum (IGV2) | Type 1 – one varicose vessel with almost the same diameter as the inflow/outflow vein (no noticeable ramifications) called the localized gastric varix  Type 2 – multiple varicose vessels with complex connecting ramifications called diffuse gastric varices |
|  | **ARAKAWA CLASSIFICATION** |
|  | Type 1 – in which a single dominant feeding channel arising from the splenic vein empties into the left renal vein through the gastric cardia and/or fundus  Type 2 – in which the vessels empty into the left renal vein in the presence of multiple feeder collaterals |
| **JAPANESE SOCIETY FOR PORTAL HYPERTENSION** | **ITALIAN ENDOSCOPIC CLASSIFICATION** |
| Gastric varices isolated at fundus/fornix (Lg f)  Gastric varices located at the cardiac (Lg-c)  Gastric varices located at both fundus and cardiac regions (Lg c-f)  Gastric varices at antrum (Lg-a)  Gastric varices at the body of stomach (Lg-b) | Type I gastric varices – all gastroesophageal varices  Type II varices – isolated gastric varices and ectopic gastric varices |
|  | **SIMPLE CLASSIFICATION GASTRIC VARICES** |
|  | Primary gastric varices – may or may not be associated with a shunt  Secondary gastric varices – developing after endoscopic band ligation-based eradication of esophageal varices. Never associated with portosystemic shunt |

**Supplementary table 2: Hemodynamic classification of gastric varices based on portal inflow/afferent system**

| **CLASSIFICATION SYSTEM** | **CLINICAL RELEVANCE** |
| --- | --- |
| **Kiyosue Classification** | In Type 1, during occlusion of collateral pathways, administered sclerosant refluxes into the gastric variceal complex and remains there because of high pressure from the portal system and leads to complete occlusion.  In Type 2, in the presence of double pathways, one of the low-pressure efferent veins functions as a draining vein once outflow is obstructed with sclerosant, resulting in efflux of material into portal circulation.  In Type 3, since the separate vein does not supply the gastric varices, during occlusion procedure, sclerosant can reflux into the portal circulation rather than the gastric variceal complex, due to which a separate embolization of this noncommunicating shunt will be required for complete variceal occlusion. |
| Type 1 – varices supplied by a single afferent gastric vein  Type 2 – multiple afferent gastric veins supply  the varices  Type 3 – single or multiple gastric veins supply the varices in presence of other gastric veins that are directly in continuation with a shunt, do not significantly contribute to variceal  formation |  |
| **Saad – Caldwell Classification** | Type 1 correlates with Sarin classification of Type 1 GOV. In this type, endoscopic therapy of cyanoacrylate glue injection usually results in complete variceal obturation in the absence of a portosystemic shunt. In the presence of portosystemic shunt, shunt embolization is the better choice.  Type 2 correlates with Sarin classification of Type 1 IGV. This type almost always requires shunt embolization, but in the absence of shunts, only a transjugular intrahepatic portosystemic shunt placement will help control rebleeding or recurrence of bleeding.  Type 3 correlates with Sarin classification of Type 2 GOV. These are usually large and complex variceal systems and almost always associated with large portosystemic shunt. Chances of recurrence of bleeding and failure to control bleeding is high and hence interventional shunt embolization of varices is a better option in this type.  In Type 4, a transjugular intrahepatic portosystemic shunt placement is most useful in controlling rebleeding or recurrence of bleeding along with local revascularization procedures. |
| Type 1 – isolated cardio-gastric varices without fundic varices  Type 1b – presence of gastrorenal shunt  Type 1a – absence of gastrorenal shunt  Type 2 – isolated fundal gastric varices without cardiac extension  Type 2b – presence of gastrorenal shunt  Type 2a – absence of gastrorenal shunt  Type 3 – High association with esophageal varices  3b – presence of gastrorenal shunt  3a – absence of gastrorenal shunt  Type 4 – High association with esophageal varices or can be fundal gastric varices without cardiac extension, but always associated with splenic / portal vein thrombosis. |  |
